# The Frequent Repetition of Doses

**Published:** 1883-12

**Authors:** John W. Martin

**Affiliations:** Sheffield


THE FREQUENT REPETITION OF DOSES. By JOHN W. MARTIN, M.D., M.Ch., Sheffield.

[Some months ago we published in full Prof. Smith's article referred to below. Dr. Martin has made such a useful compilation of the several matters of interest contained in the article, that we reproduce it for the benefit of our readers]:

The following table of drugs, and their method of exhibition, has been compiled from a recent article in the *New York Medical Journal* by Dr. A. A. Smith, Professor of Materia Medica in Bellevue Hospital Medical College. The subject is one of great interest to practical men. The compiler has frequently found marked benefit to follow a change of dosage, in the direction of smaller amounts and greater frequency of repetition.

| DRUG. | DOSAGE. | DISEASE. |
|---|---|---|
| Chlorate of potash. | Gr. j. every half hour. | Scarlet fever, diptheria, tonsillitis, etc. |
| Croton chloral. | Gr. ss. Ditto. | Neuralgia. |
| *Note.*—Solution of a grain to the drachm is almost tasteless. | | |
| Salicylate of sodium. | Gr. ij. hourly or half-hourly. | Urticaria (acute). |
| *Note.*—Useless in chronic urticaria or that produced by balsam copaiba. | | |
| Fowler's solution. | Gr. ss. half-hourly for 6 or 8 doses. | Vomiting after a debauch. Drunkard's morning vomiting. Vomiting of pregnancy. |
| Liquor potassii arsenitis. | Ditto. | Ditto |
| Jaborandi (fl. ext.), with or without digitalis. | Min. v.-x. hourly or half-hourly. | Produces copious perspiration, without causing any unpleasant heart symptoms. |
| Sulphate of atropia. | A teaspoonful of a solution of one 1-100th of a grain in a tumbler of water hourly or half-hourly. | Supposed croup, which is usually false croup of a reflex origin. |
| *Note.*—If the child's face flushes, and physiological action of drug appears, frequency of dose to be diminished. | | |
| Bromides. | Grs. j -ij. every 10 or 15 minutes. | Nervous disturbances and transitory disorders of children. |
| Tr. Chamomille. | Min. j. every 15 or 20 minutes. | Insomnia of nervous, excitable sensitive children. |
| Vin. ipecac. | Gtt. j. every 15 or 20 minutes | Vomiting or diarrhœa. |
| Calomel. | 1-60th of a grain every hour for 10 or 12 hours. | Relieves the headache of syphilis occurring at night. |
| *Note.*—It is not to replace the iodides. This is Trousseau's method. The author found it successful by the second or third night. | | |
| Calomel. | Drachm j., every 10 or 15 minutes, of a solution of gr. j. to the pint of water. | Vomiting or regurgitation of food in the case of nursing children. |
| Hyd. c. cretâ. | Gr. 1-24th every 15 or 20 minutes. | Vomiting and non-inflammatory diarrhœa of children. |
| Hyd. bichloridi. | Drachm j., every hour, of a solution of gr. j. to the quart of water. | Diarrhœa in children, with mucous passages and inflammatory symptoms. |
| Tartar emetic. | Drachm j., every half-hour, of a solution of gr. j. to the quart of water. | Wheezing and cough of slight bronchitis in children. |
| Tr. nucis vomicæ. | Gtt. j. every 10 minutes. | Sick headache, not of a neurotic origin. |
| *Note.*—To be given immediately or soon after meals. | | |
| Cantharides. | Min. j. every hour. | Vesical catarrh. |
| Tr. digitalis. | Gtt. j. hourly or half-hourly. | Organic heart disease where digitalis is indicated. |
| *Note.*—Gives greater relief without liability to ill effects. | | |
| Castor oil. | Gtt. v. every hour in water, with sugar and gum. | Diarrhœa of children, with straining, slight inflammation and passage of jelly-looking matter, but not true dysentery. |
| Tr. of pulsatella. | Min. ij. every hour. | Orchitis, epididymitis and dysmenorrhœa, not of a membranous, obstructive or neurotic character. |
| *Note.*—Affords greater relief than any other method of treatment. | | |

| DRUG. | DOSAGE. | DISEASE. |
|---|---|---|
| Calabar bean. | Gr. 1-50th every half-hour for 6 or 8 doses. Then stop for 3 hours, and again similarly repeat. | Flatulence, fluttering and palpitation of the menopause. |
| Ergot (fl. ext.). | Min. j every half-hour for 5 or 6 hours. | Amenorrhœa, not due to anæmia. To be given the day before and again on the day on which it should occur. |
| Aconite (tr.). | Min. ⅓ to ½ very 15 minutes. | Febrile disturbance, cold in the head, cardiac hypertrophy with palpitation, severe headache, disturbances of nervous system due to increased force of heart-beat. |
| *Note.*—Effects must be watched. Dose regulated accordingly. | | |
| Hamamelis (tr.), | Min. ij. every half-hour. | Controls hæmorrhages. |
| Belladonna (tr.). | Min. j. every half-hour for 8 or 10 doses; then cease. | Nasal catarrh and bronchitis with free secretion. Pulmonary œdema, with failure of heart-power. |
| *Note.*—Retards exudation of serum; overcomes failures of heart power. | | |
| Chloride of ammonium. | Grs. ij., combined with min. x-xv. of tr. cubebs every half-hour. | Acute pharyngitis, superficial inflammations of the other tissues of the throat. |
| *Note.*—If throat inflammations are due to the gouty diathesis, add to mixture min. x. of the ammoniated tincture of guaiac, and give every hour. | | |
| Citrate of caffeine. | Gr. j. every half-hour. | Headache of migraine. |
| Gelseminum (tr.). | Min. iij. every half-hour. | Neuralgias about the head and face. |
| Guarana (fl. ext.). | Min. xv. every 15 minutes for 4 doses; then, if no relief, increase to min. xxx. | Headaches, especially those which are periodical and not of malarial origin. |

—*Med. Press and Circular.*

